# Prolonged Administration of *Rudgea viburnoides* (Cham.) Benth. Prevents Impairment of Redox Status, Renal Dysfunction, and Cardiovascular Damage in 2K1C-Hypertensive Rats by Inhibiting ACE Activity and NO-GMPC Pathway Activation

**DOI:** 10.3390/pharmaceutics13101579

**Published:** 2021-09-28

**Authors:** Fernanda Viana Paulin, Rhanany Alan Calloi Palozi, Bethânia Rosa Lorençone, Arthur Ladeira Macedo, Lucas Pires Guarnier, Cleide Adriane Signor Tirloni, Paulo Vitor Moreira Romão, Arquimedes Gasparotto Junior, Denise Brentan Silva

**Affiliations:** 1 Laboratório de Produtos Naturais e Espectrometria de Massas (LaPNEM), Faculdade de Ciências Farmacêuticas, Alimentos e Nutrição (FACFAN), Universidade Federal do Mato Grosso do Sul, Campo Grande 79070-900, Brazil; fernanda@movecor.com.br (F.V.P.); arthur.ladeira@ufms.br (A.L.M.); 2 Laboratório de Farmacologia Cardiovascular (LaFaC), Faculdade de Ciências da Saúde, Universidade Federal da Grande Dourados, Dourados 79825-070, Brazil; palozirhanany@gmail.com (R.A.C.P.); bethaniarosalorencone@hotmail.com (B.R.L.); rannier.andrade@outlook.com (L.P.G.); cleide.4132@gmail.com (C.A.S.T.); paulovitor_moreiraromao@hotmail.com (P.V.M.R.); arquimedesjunior@ufgd.edu.br (A.G.J.)

**Keywords:** antihypertensive, antioxidant, cardioprotective, diuretic, renoprotective, iridoid

## Abstract

*Rudgea viburnoides* is widely found in the Brazilian Cerrado, and commonly used in Brazilian folk medicine. In this study, we evaluated the effects of prolonged administration of the aqueous extract from *R. viburnoides* leaves (AERV) on impaired redox status, renal dysfunction, and cardiovascular damage in 2K1C hypertensive rats, as well as its chemical composition by LC-DAD-MS. Renal hypertension (two kidney, one-clip model) was surgically induced in male Wistar rats and AERV (30, 100 and 300 mg/kg) was administered orally five weeks after surgery for 28 days. Renal function was assessed and urinary electrolytes, pH, and density were measured. Electrocardiography, blood pressure and heart rate were recorded. Cardiac and mesenteric vascular beds were isolated for cardiac morphometry and evaluation of vascular reactivity, and aortic rings were also isolated for measurement of cyclic guanosine monophosphate levels, and the redox status was assessed. Prolonged treatment with AERV preserved urine excretion and electrolyte levels (Na^+^, K^+^, Ca^2+^ and Cl^−^), reversed electrocardiographic changes, left ventricular hypertrophy and changes in vascular reactivity induced by hypertension, and reduced blood pressure and heart rate. This effect was associated with a positive modulation of tissue redox state, activation of the NO/cGMP pathway, and inhibition of the angiotensin-converting enzyme. Glycosylated iridoids, chlorogenic acids, glycosylated triterpenes, *O*-glycosylated flavonols, and triterpenoid saponins were annotated. AERV showed no acute toxicity in female Wistar rats. Therefore, AERV treatment reduced the progression of cardiorenal disease in 2K1C hypertensive rats, which can be involved with an important attenuation of oxidative stress, angiotensin-converting enzyme inhibition, and activation of the NO/cGMP pathway.

## 1. Introduction

Hypertension, a multifactorial disease characterized by sustained elevation of the blood pressure (BP) levels, affects 1.13 billion people around the world. It is a risk factor for cardiovascular diseases, including chronic kidney disease, atherosclerosis, stroke, and heart attack [1,2].

According to the 2017 Guidelines for Arterial Hypertension Management in Primary Health Care in Portuguese Language Countries, Brazil has 17 million hypertensive patients, and cardiovascular disease (CVD) is the leading cause of death. The outlook is that by 2025, this number is expected to grow 80% mainly due to smoking, sedentary lifestyle, obesity, poor diet, and high alcohol consumption [3].

The recommended drugs to treat hypertension include beta blockers, calcium channel blockers, diuretics, and renin-angiotensin system inhibitors. In addition, changes in lifestyle are recommended, such as regular physical exercise, healthy eating, and body weight control. These measures are mainly aimed at preventing cardiovascular complications and metabolic changes due to hypertension [4]. Allied to this, the uses of alternative therapies have increased, mainly in underdeveloped or developing countries, such as the application of medicinal plants [5].

Medicinal plants have been used by populations over several generations to treat several disorders, such as hypertension [6]. In this context, *Rudgea viburnoides* (Cham.) Benth. (Rubiaceae) is a widely known species in the Brazilian Cerrado, popularly named as ‘congonha de bugre’, ‘congonha’, ‘bugre’, and ‘porangaba’ [7,8]. It is traditionally used as hypotensive, antiarrhythmic, diuretic, blood depurative, in slimming diets, and to treat kidney and bladder infections [9].

From *R. viburnoides*, chemical and pharmacological studies are still scarce. Preclinical studies have reported nephroprotective [10], diuretic [11], lipid-lowering, and anti-inflammatory effects [12]. In addition, no toxic effects were reported after acute administration of 5000 mg/kg of the ethanolic extract obtained from the leaves of *R. viburnoides* in rats and mice [11]. Among its main compounds, there are chlorogenic and caffeic acids, flavonoids [8,12], triterpenes, and saponins [13].

Considering that the *R. viburnoides* is widely used in traditional medicine to treat hypertension, it is interesting and important to investigate its abilities to promote cardioprotective effects in hypertensive rats, as well as expand the chemical information about its composition. Therefore, the aim of this study was to evaluate the aqueous extract obtained from leaves of *R. viburnoides* (AERV) on impairment of redox status, renal dysfunction, and cardiovascular damage in 2K1C-hypertensive rats.

## 2. Materials and Methods

### 2.1. Drugs and Reagents

Xylazine, ketamine hydrochloride (Syntec, São Paulo, SP, Brazil), and heparin (Hipolabor, Belo Horizonte, MG, Brazil) were used in the experiments. The reagents acetylcholine chloride, angiotensin II, indomethacin, metoprolol, NaCl, MgSO_4_, CaCl_2_, dextrose, phenylephrine, KH_2_PO_4_, ethylenediaminetetraacetic acid, 2′,7′-dichlorofluorescein-diacetate, 5,5′-dithiobis, sodium nitroprusside, bovine serum albumin, KCl, NaHCO_3_, reduced glutathione, Tris-HCl, xylenol orange, and ethylenediaminetetraacetic acid were purchased from Sigma–Aldrich (St. Louis, MO, USA). For LC-DAD-MS analyses, methanol, acetonitrile, and formic acid were HPLC grade and purchased from J.T. Baker (Phillipsburg, NJ, USA).

### 2.2. Plant Material and Extraction

*Rudgea viburnoides* leaves were harvested at the Private Natural Heritage Reserve of the Federal University of Mato Grosso do Sul (RPPN/UFMS) (S20°30′32.0′′–W54°36′57.7′′). Dr. Flávio Macedo Alves identified the plant, and a voucher was deposited at CGMS Herbarium under number 74319. This study is registered at National Genetic Heritage Management System (SisGen, access date 25 August 2021) of the Brazilian ministry of environment under number ACD1712.

The leaves were dried (50 °C for 48 h), powdered (knife mill), and extracted by accelerated solvent extractor Dionex™ ASE™ using hexane and subsequent water, with a plant–solvent ratio of 1:30. The following parameters were applied for extraction: three cycles of 4 min with hexane (100 °C, purge of 100 s and rinse of 60%) to degrease the sample and two cycles of 4 min using distilled water (100 °C, rinse of 100% and purge of 60 s) to obtain the aqueous extract. This extract was lyophilized to obtain the aqueous extract from leaves of *R. viburnoides* (AERV) with yielded of 9.9%. AERV was maintained at −20 °C until the experiments. The hexane and AERV extracts were obtained with the yielded of 10% and 26.7%, respectively.

### 2.3. Chemical Analyses by LC-DAD-MS

AERV was solubilized in methanol and ultrapure water 1:1 (*v*/*v*) at 2 mg/mL. This solution was filtered on syringe filter (Millex, PTFE, 0.22 μm, Millipore) and 5 μL was injected on the chromatographic system. For the LC-DAD-MS analyses, an UFLC Shimadzu Prominence chromatographic system coupled to a diode array detector (DAD) and a mass spectrometer (MicrOTOF-Q III, Bruker Daltonics, Billerica, MA, USA) was used. The mass spectrometer was provided by an electrospray ion source and analyzers quadrupole and time-of-flight. A Kinetex C18 (2.6 μm, 100 A, 150 × 2.1 mm) chromatographic column (Phenomenex, Torrance, CA, USA) was used; the flow rate was 0.3 mL/min and the chromatographic column was maintained at 50 °C in the analyses. All the LC and MS parameters were the same described by Younis et al. [14], and negative and positive ion modes were applied. The molecular formulae were determined based on the accurate mass (±7 ppm) and mSigma below 30. The negative mode chromatogram was used to calculate the relative area of the annotated compounds.

### 2.4. Ethnopharmacological Studies

#### 2.4.1. Animals

Male and female Wistar rats (13–14 weeks) were acquired from the Federal University of Mato Grosso do Sul (UFMS). These animals (randomized and housed) were maintained at 22 ± 2 °C under a 12:12 h light:dark cycle and ad libitum access to food and water. The Ethics Committee in Animal Experimentation from UFMS previously approved the procedures (protocol number 947/2018). The procedures were conducted in accordance with the Guidelines for the Care and Use of Laboratory Animals as adopted and promulgated by the United States National Health Institute [15].

#### 2.4.2. Acute Toxicity

Acute toxicity was evaluated in female rats according to protocol 425 from the Organization for Economic Co-operation and Development [16]. The extract AERV (three single doses at 30, 300, and 2000 mg/kg) or water (1 mL/kg) were administered to female rats (*n* = 8, oral gavage). Following administration, animals were closely observed during the first 24 h and daily for 14 consecutive days. Mortality, body weight gain, and food and water consumption were daily observed and recorded. The animal behavior was also individually observed as described by Malone and Robichaud [17]. On the fifteenth day, the rats were euthanized by isoflurane anesthesia followed by exsanguination. Heart, lung, spleen, liver, kidneys, uterus, and ovaries were removed, weighed, and macroscopically examined. Heart, liver, and kidney samples were fixed in 10% buffered formalin. Samples were sectioned and stained with hematoxylin/eosin (H&E). Analyses were performed under light microscopy (40×) (Olympus CX 31) (Supplementary Material).

#### 2.4.3. Hypertension Induction (Goldblatt Model; Two Kidneys, One Clip; 2K1C)

After the anesthesia by intraperitoneal injection of ketamine at 100 mg/kg and xylazine 20 mg/kg, the male rats were submitted to laparotomy, the left renal artery was isolated, and a silver clip (1.5 mm lumen) was placed to partially limit blood flow [18]. Systolic blood pressure (SBP) was weekly measured by the tail-cuff method. Only animals with SBP above 140 mm Hg were used in experiments.

#### 2.4.4. Experimental Design

Six experimental groups with 8 to 10 animals were randomized after five weeks of surgery. For 28 days, the animals were orally treated with vehicle (filtered water; positive control), metoprolol (MPL, 20 mg/kg), and AERV at doses 30, 100, or 300 mg/kg. The negative control (NC) was the Sham-operated rats that were treated with the vehicle.

#### 2.4.5. Renal Function

The renal function was evaluated according to methods previously described [19]. After the treatments, the animals were placed in metabolic cages. The urinary volume was measured and expressed as mL/100 g of body weight. A digital pH meter (Q400MT; Quimis Instruments, Diadema, SP, Brazil) was applied to determine the pH. A refractometer NO107 (Nova Instruments, Piracicaba, SP, Brazil) was used to estimate the density. For the determinations of sodium (Na^+^), potassium (K^+^), chloride (Cl^−^), and calcium (Ca^2+^) levels, an automatic biochemistry analyzer (COBAS INTEGRA 400 plus; Roche^®^) was used.

#### 2.4.6. Electrocardiography

On the morning of the twenty-ninth day, all animals underwent electrocardiography (ECG). The animals were anesthetized (ketamine 100 and xylazine 20 mg/kg; intramuscularly). The electrodes were positioned on the animal’s two forelimbs and two hindlimbs. After 5 min to acclimatize, ECG waves were recorded for more 5 min. The segments (ms): PR, QRS, QT, and QTc wave amplitudes (mV): P, Q, R, and S were recorded. A 12-lead ECG recorder was applied to record electrocardiography (WinCardio, Micromed, Brasilia, Brazil).

#### 2.4.7. Blood Pressure (BP) Assessment

After electrocardiography, all rats received a heparin injection (50 IU; subcutaneously). The carotid artery (left) was coupled to a pressure transducer (PowerLab^®^ recording system—program Chart, v 4.1; all from ADI Instruments, Castle Hill, Australia), which was initially isolated and cannulated. Then, the following parameters were recorded for 20 min: diastolic blood pressure (DBP), systolic blood pressure (SBP), and mean arterial pressure (MAP).

#### 2.4.8. Biochemical Parameters

After the BP recording, from the carotid artery (left) the blood samples were obtained, and by centrifugation (1500× *g*, 10 min) the serum samples were acquired. An automated biochemical analyzer (Roche Cobas Integra 400 plus) was applied to measure Na^+^, K^+^, urea, and creatinine levels. The serum angiotensin converting enzyme (ACE) activity was measured according by Santos et al. [20].

#### 2.4.9. Mesenteric Vascular Beds (MVBs) Reactivity

After blood collection and before euthanasia, the mesenteric vascular beds (MVBs) were obtained as described by McGregor [21]. The organs were placed in an organ bath and perfused at 6 mL/min with PSS (119 mM NaCl, 4.7 mM KCl, 2.4 mM CaCl_2_, 1.2 mM MgSO_4_, 25.0 mM NaHCO_3_, 1.2 mM KH_2_PO_4_, 11.1 mM dextrose, and 0.03 mM EDTA) at 37 °C and gassed with 95% O_2_/5% CO_2_. A pressure transducer coupled to a PowerLab^®^ recording system (Chart, v 4.1; all from ADI Instruments, Castle Hill, Australia) was applied to detect changes in the perfusion pressure (PP; mmHg). The vascular reactivity was determined for phenylephrine (Phe; 0.1, 0.3, and 1 nmol; 10–30 μL), sodium nitroprusside (SNP; 1, 3, and 10 pmol; 10–30 μL), and acetylcholine (ACh; 1, 3, and 10 pmol; 10–30 μL).

#### 2.4.10. Relative Weight and Histopathological Analysis of the Heart and Left Ventricle Morphometry

After euthanasia, removal of the heart was performed, and subsequently it was longitudinally sectioned and cleaned. The relative weight of the heart was determined by formula WT% = absolute organ weight × 100/body weight. The cardiac tissue (a part) was also placed in 10% buffered formalin. Tissue sections (5 mm) were acquired, stained with hematoxylin and eosin, and analyzed by a light microscope (Olympus CX 31, Tokyo, Japan). Motic Images plus 2.0 software was applied to image data acquisition and analyses.

#### 2.4.11. Cardiac and Vascular Redox Status

After euthanasia, parts of the tissue heart, aorta, and right kidney were removed and homogenized in K^+^ phosphate buffer (0.1 M, pH 6.5). Superoxide dismutase (SOD) was evaluated according to Gao et al. [22]. Lipid peroxidation (LPO) was measured according to Jiang et al. [23]. Catalase (Cat) was measured according to Beers and Sizer [24]. The results were expressed by the amount of protein [25].

#### 2.4.12. Cyclic Guanosine Monophosphate (cGMP) Determination

The role of AERV on cyclic guanosine monophosphate (cGMP) levels was evaluated according to Estancial et al. [26]. Aortic rings from 2K1C rats (2–3 mm; *n* = 5) were removed and placed in an organ bath with Krebs–Henseleit solution (composition in mm: 4.7 KCl, 117 NaCl, 1.2 MgSO_4_, 2.5 CaCl_2_, 1.2 KH_2_PO_4_, 25 NaHCO_3_, and 11 glucose) at 37 °C with 95% O_2_ and 5% CO_2_. Aortic rings were incubated with SNP (10 μm), or AERV at the concentrations of 0.001, 0.003, and 0.01 mg/mL for 15 min in the presence or absence of ODQ (100 μm, 30 min). From tissues samples the supernatants were collected after their removal and homogenization. The intracellular cGMP levels were determined as described by the manufacturer (Cayman Chemical Cyclic GMP EIA kit, Ann Arbor, MI, USA). All experiments were performed in triplicate.

### 2.5. Statistical Analyses

Data were analyzed for normal distribution and homogeneity of variance. For the statistical analysis, one-way analysis of variance (ANOVA) and Bonferroni post hoc test were applied. The significance level was set at 95% (*p* < 0.05) and results were expressed as mean ± standard error of the mean (S.E.M.).

## 3. Results

### 3.1. Chemical Constituents from AERV

**Figure 1:**
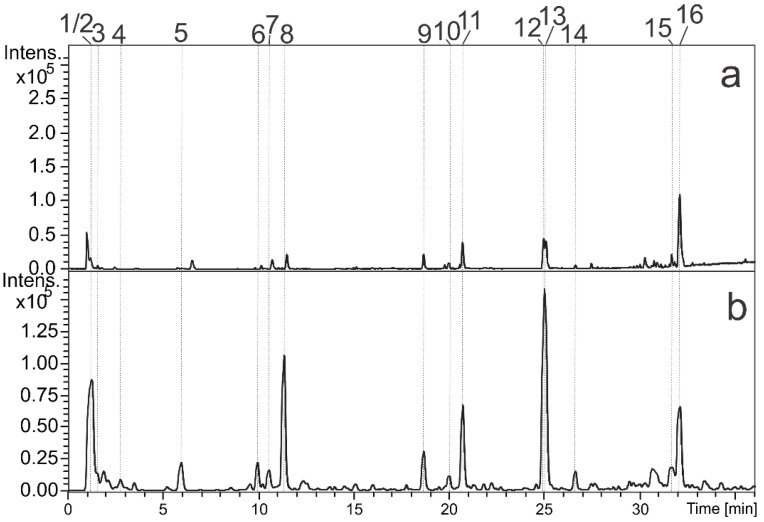
Base peak chromatograms from leaves aqueous extract of *Rudgea viburnoides* (AERV) obtained in positive (**a**) and negative ion modes (**b**).

LC-DAD-MS was applied to analyze the aqueous extract from *R. viburnoides* (AERV) and its constituents were annotated and summarized on Figure 1 and Table 1. The annotation of compounds was based on spectral data (UV, MS, and MS/MS) compared with published data. Further, some compounds were confirmed by the injection of authentic standard.

Peaks 1, 2, and 3 presented deprotonated ions at *m/z* 191.0534, 341.1095, and 191.0209. They were compatible to molecular formulae C_7_H_12_O_6_, C_12_H_22_O_11_, and C_6_H_8_O_7_, and they were putatively annotated as quinic acid (1), di-*O*-hexoside (2), and citric acid (3), respectively. Compounds 5 and 7 showed UV spectra similar to the observed for caffeic acid chromophores (λ_max_ ≈ 299 and 325 nm) [27]. The fragment ions at *m/z* 191 and 179 were relative to quinic acid and caffeic acid, and the relative abundance of ion *m/z* 179 was used to suggest the esterification position of quinic acid [28]. Thus, the metabolites 5 and 7 were identified as 3-*O-E*-caffeoyl quinic acid and 5-*O-E*-caffeoyl quinic acid and they were confirmed by injection of authentic standards.

Peaks 6 and 8 did not present absorption on UV and revealed deprotonated ions at *m/z* 431.1195 (C_18_H_24_O_12_) and 413.1084 (C_18_H_22_O_11_). The losses of hexosyl (162 *u*) and subsequently of a water molecule (18 *u*), as well as the losses of a cetene (C_2_H_2_O, 42 *u*) and carbon dioxide (CO_2_, 44 *u*), suggested for both compounds the presence of hexosyl, acetyl, and carboxylic acid groups in the structures of 6 and 8, for example, the fragment ions *m/z* 251 [M-H-hexosyl-H_2_O]^−^, 165 [M-H-hexosyl-H_2_O-C_2_H_2_O-CO]^−^, and 191 [M-H-hexosyl-H_2_O-C_2_H_2_O]. They were putatively annotated as the iridoids asperulosidic acid and asperuloside, which showed similar spectral data reported for them and they were already identified in Rubiaceae family [29,30,31].

The characteristic UV absorption bands of 9 (265 and 355 nm) and 11 (265 and 346 nm) suggested that these compounds are flavonols [27]. Compounds 9 and 11 displayed the deprotonated ion at *m/z* 609.1472 and 593.1521, indicating molecular formulas of C_27_H_30_O_16_ and C_27_H_30_O_15_, respectively. Both compounds showed a loss of 308 *u* that is relative to hexosyl and deoxyhexosyl substituents, and its aglycones showed *m/z* 301 (9) and 285 (11). In this way, 9 and 11 were putatively identified as *O*-hexosyl-deoxyhexosyl quercetin and *O*-hexosyl-deoxyhexosyl kaempferol [32].

The isomers 12 and 13 showed ions at *m/z* 681.3823 and 681.3832 [M-H]^−^ and suggested the molecular formula of C_36_H_58_O_12_. The fragmentation pattern of these compounds was similar; the product ion *m/z* 519 was yielded from the loss of a hexosyl group (162 *u*). The ions with *m/z* 407 and 207 are relative to a retro Diels–Alder (RDA) cleavage (double bound on C12, ring C), followed by dehydration. The ion *m/z* 407 is relative to rings D and E moiety *O*-linked to the H group, while the *m/z* 207 represents the rings A and B. In this way, 12 and 13 were identified as trachelosperosides B-1 or E-1 [33,34]. These compounds were already reported from *R. viburnoides* by Young et al. [13].

Finally, compounds 15 and 16 were annotated as triterpenoid saponins. Compound 16 (*m/z* 955.4914 [M-H]^−^, C_48_H_76_O_19_) showed sequential losses of 162 and 176 *u*, suggesting the substituents hexosyl and glucuronyl, and these losses yielded the fragment ion *m/z* 455 (C_30_H_47_O_3_^−^) relative to aglycone. Compound 15 (*m/z* 1087.5303 [M-H]^−^, C_53_H_84_O_23_) presented similar to 16, but with an additional substituent *O*-pentosyl and the data suggested that 15 and 16 are bisdesmosidic saponins [35].

### 3.2. Toxicological Evaluations

No changes in behavior nor deaths were observed in the animals during the observation period (14 days). However, the treatment with AERV (all doses) showed a significant reduction on feed consumption and body weight gain in the animals. Regarding the relative organ weight, notable differences were not observed for the rats treated with AERV compared to the control animals. The heart, lung, liver, ovaries, uterus, spleen, and kidneys were analyzed, and no significant changes were observed, as well as no histopathological changes in heart, liver, or kidneys (Supplementary Material).

### 3.3. Effects on Urinary Volume and Renal Electrolyte Excretion

None of the experimental groups had significant differences in urinary volume excreted on the first day of treatment (Table 2), although a remarkable reduction in renal excretion of Na^+^, Ca^2+^, and Cl^−^ were observed for the animals of negative control and treated with AERV 30 and 100 mg/kg compared to Sham-operated animals. Interestingly, the animals treated with AERV (300 mg/kg) or MPL revealed urinary Na^+^, Ca^2+^, and Cl^−^ values similar to those observed for Sham-operated animals. In addition, the urinary density for the negative control group was lower than the observed for the Sham-operated group. All other parameters evaluated were not changed by any of the treatments performed.

The effects of the AERV treatment on the renal function on the seventh day of treatment are described in Table 3. A decrease in urinary excretion was observed for the negative control group and AERV-treated rats (30, 100, and 300 mg/kg) compared to the Sham-operated animals. In addition, an increase in urinary density was observed for the negative control and AERV 30 and 100 mg/kg groups. On the other hand, an important reduction in urinary density was determined for the animals treated with MPL or AERV at 300 mg/kg compared to the negative control group. The elimination of electrolytes or the urinary pH were not significantly changed from the evaluated treatments.

The urinary volume and renal excretion of Na^+^, Ca^2+^, and Cl^−^ were reduced in negative control animals on the fourteenth day of treatment. The renal elimination of electrolytes and the urinary volume for the AERV treatment at doses of 30 and 100 mg/kg were similar to the negative control (Table 4). At the highest dose (300 mg/kg) of AERV treatment, no significant changes in urine elimination, nor Cl^−^, Ca^2+^, and Na^+^ urinary levels were observed when compared to the Sham group, but significant reductions in urinary levels of Cl^−^ and Ca^2+^ were observed for the MPL-treated group (Table 4).

The effects of prolonged AERV treatment (on the twenty-first day) on the different urinary parameters are summarized in Table 5. On that day, the animals of the negative control group showed the lowest urinary volume and electrolyte excretion levels (K^+^ and Ca^2+^). The animals treated with MPL also exhibited reduction in urinary volume and renal excretion of Ca^2+^ and Cl^−^ levels. In addition, the AERV treatments at doses of 30 and 100 mg/kg significantly reduced the urinary volume compared with animals of the Sham-operated group. Otherwise, the renal function was preserved in hypertensive rats treated with AERV (300 mg/kg), and these effects were similar with those observed by Sham-operated animals. The urinary density observed for the negative control rats, MPL, or AERV (30 and 100 mg/kg) were significantly higher than the animals of the Sham or AERV (300 mg/kg) groups.

The values of the different urinary parameters on the 28th day of treatment were summarized in Table 6. The urinary excretion was reduced in negative control group, as well as the levels of Cl^−^, Ca^2+^, and Na^+^. The MPL treatment also showed an impaired urinary volume, and lower urinary Ca^2+^, and Cl^−^ levels than rats in the Sham group. The urinary volume and renal elimination of Ca^2+^, accompanied by normalization of urinary elimination of Na^+^, K^+,^ and Cl^−^, were reduced with the AERV treatment at 30 and 100 mg/kg (Table 6). Nonetheless, the animals treated with AERV (300 mg/mL) revealed a urinary volume and electrolyte excretion similar to those observed for Sham-operated rats. Additionally, the urinary density remained significantly increased for the negative control or AERV at dose of 30 mg/kg groups (Table 6).

### 3.4. Effects on Electrical Cardiac Activity

**Figure 2:**
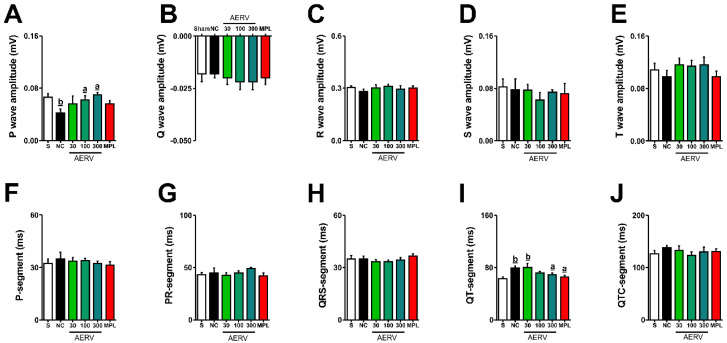
Electrocardiographic quantitative data from Sham (S) or 2K1C-hypertensive rats treated with AERV (30, 100, and 300 mg/kg), MPL (20 mg/kg), or vehicle (NC). P (**A**), Q (**B**), R (**C**), S (**D**), and T-wave amplitudes (**E**) and P (**F**), PR (**G**), QRS (**H**), QT (**I**), and QTC-segments (**J**) are shown. The values are expressed as mean ± S.E.M. In each group, 8 to 10 animals were used, and the comparisons were performed with the negative control (NC, ^a^ *p* < 0.05) or Sham-operated rats (^b^ *p* < 0.05) applying one-way ANOVA followed by Bonferroni’s test. AERV: aqueous extract from *Rudgea viburnoides*; MPL: metoprolol; NC: negative control.

Figure 2A–J shows quantitative electrocardiography data for Sham-operated and 2K1C-hypertensive rats treated with AERV (30, 100, and 300 mg/kg), MPL (20 mg/kg), or vehicle. We did not observe any significant changes in electrocardiographic characteristics of amplitude of the Q, R, S, and T waves, or the P, PR, QRS, and QTC segments in any of the experimental groups. However, the hypertensive rats, treated only with the vehicle, presented electrocardiographic changes, including significant P wave shortening and prolongation of the QT segment (Figure 2A–J). However, these alterations were prevented by AERV treatment (mainly at doses of 100 and 300 mg/kg) and MPL. The values of all parameters of that were evaluated AERV or MPL treatments were statistically similar to Sham-operated rats.

### 3.5. Effects on Blood Pressure and Heart Rate

**Figure 3:**
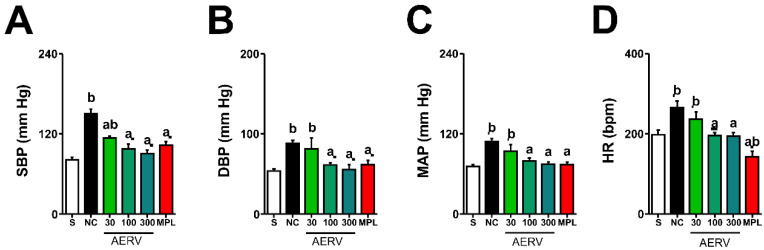
Prolonged oral administration of AERV obtained from *Rudgea viburnoides* reverses changes in blood pressure and heart rate induced by 2K1C hypertensive rats. SBP (**A**), DBP (**B**), MAP (**C**), and HR (**D**) are shown. The values are expressed as mean ± S.E.M. In each group, 8 to 10 animals were used, and the comparison between negative control (NC, ^a^ *p* < 0.05) or Sham-operated rats (^b^ *p* < 0.05) were performed applying one-way ANOVA followed by Bonferroni’s test. AERV: aqueous extract from *Rudgea viburnoides*; MPL: metoprolol; NC: negative control; S: Sham-operated group.

Figure 3 shows the effects of prolonged treatment with AERV or MPL on different hemodynamic parameters. Hypertensive animals treated only with the vehicle showed a significant increase in MAP, SBP, DBP, and HR levels. Prolonged oral treatments with AERV (at doses of 100 and 300 mg/kg) reduced MAP, SBP, DBP, and HR values compared to negative control group. Additionally, a reduction of the HR was confirmed in the MPL treatment compared to the Sham-operated animals.

### 3.6. Effects on Serum Parameters

A significant increase in urea and creatinine levels were observed in the negative control rats, as well as with ARV-treatment at dose 30 mg/kg when compared with Sham-operated animals. Further, the AERV treatment with doses of 100 and 300 mg/kg, and MPL prevented an increase in serum urea and creatinine levels, revealing similar values for the Sham-operated group (Table 7), although the serum levels of Na^+^ and K^+^ were similar in all experimental groups (Table 7).

After 28 days of treatment with vehicle (negative control) or AERV at 30 mg/kg, all 2K1C hypertensive rats showed that the plasma ACE activity increased. Otherwise, 2K1C-hypertensive rats treated with AERV at 100 and 300 mg/kg or MPL exhibited an ACE activity reduced, showing values close to the Sham-operated group (Table 7).

### 3.7. Effects on MVBs Reactivity

**Figure 4:**
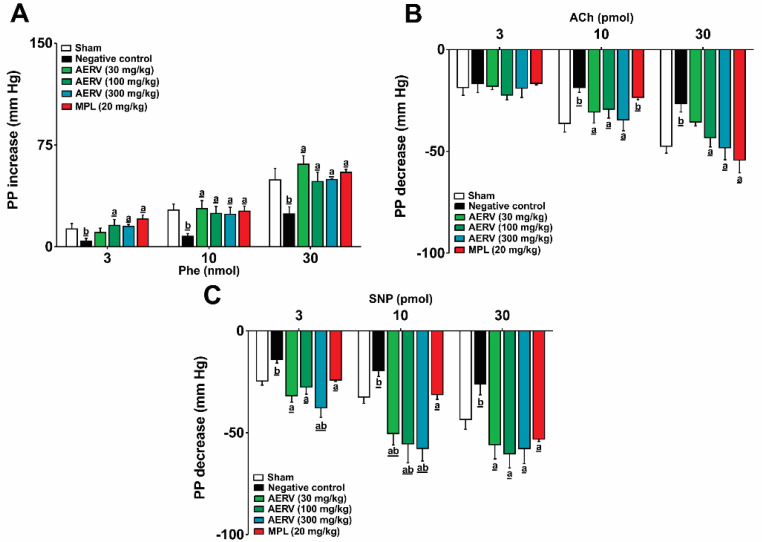
Effects of the prolonged oral administration of AERV obtained from *Rudgea viburnoides* on mesenteric vascular reactivity of 2K1C-hypertensive rats. Effects of phenylephrine (**A**), acetylcholine (**B**), and sodium nitroprusside (**C**) are shown. The values are expressed as mean ± S.E.M. In each group, 8 to 10 animals were used, and the comparison between negative control (NC, ^a^ *p* < 0.05) or Sham-operated rats (^b^ *p* < 0.05) were performed applying one-way ANOVA followed by Bonferroni’s test. ACh: acetylcholine; AERV: aqueous extract from *R. viburnoides;* MPL: metoprolol; NC: negative control; Phe: phenylephrine; PP: perfusion pressure; SNP: sodium nitroprusside.

In the negative control group, Phe administration induced a vasoconstrictive effect in the MVBs lower (~50%) compared to Sham-operated rats (Figure 4A). The negative control group also showed a lower vasodilatory response to ACh and SNP than normotensive rats (Figure 4B,C). The prolonged treatment with AERV (at all doses) prevented all changes, and the pattern of SNP-response was remarkable higher when compared to Sham-operated animals. Furthermore, the response with the AERV-treatment was significantly higher than MPL treatment.

### 3.8. Effects on Cardiac Morphology

**Figure 5:**
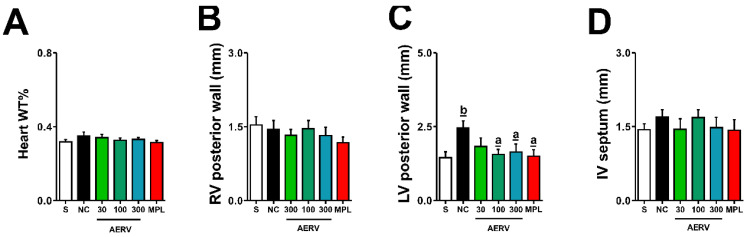
Effects of the AERV treatment on heart histopathological changes induced by 2K1C-hypertensive rats. WT% of the heart (**A**), and morphometric data of right ventricle posterior wall (**B**), left ventricle posterior wall (**C**), and interventricular septum (**D**) are shown. The values are expressed as mean ± S.E.M. In each group, 8 to 10 animals were used, and the comparison between negative control (NC, ^a^ *p* < 0.05) or Sham-operated rats (^b^ *p* < 0.05) were performed applying one-way ANOVA followed by Bonferroni’s test. AERV: aqueous extract from *Rudgea viburnoides*; MPL: metoprolol; NC: negative control; S: Sham-operated group.

The relative weight of the heart (WT%) and morphometric measures of the right ventricle (RV), left ventricle (LV), and interventricular septum (IV) are shown in Figure 5A–D. The LV posterior wall thickness (1.45 ± 0.20 vs. 2.46 ± 0.23 mm) was increased in the negative control group compared with Sham-operated animals. However, the thickness of the LV in 2K1C-hypertensive rats were reduced with the AER and MPL treatments, showing similar values observed for normotensive animals.

### 3.9. Effects on Cardiac and Vascular Redox Status

The effects on tissue redox status from the treatments were evaluated and the results are summarized in Table 8. The lipid peroxidation contents were increased in renovascular hypertension at approximately 60% and 30% in the heart and aorta samples, respectively, when compared to Sham-operated group. Besides, in negative control rats, the Cat and SOD activities were decreased, while in AERV-treatment (100 and 300 mg/kg) the SOD and Cat activities were increased. Additionally, the lipid peroxidation was prevented in all evaluated tissues, but AERV and MPL treatment were not reversed the changes in heart and vascular tissues.

### 3.10. Effects on Intracellular cGMP Levels

**Figure 6:**
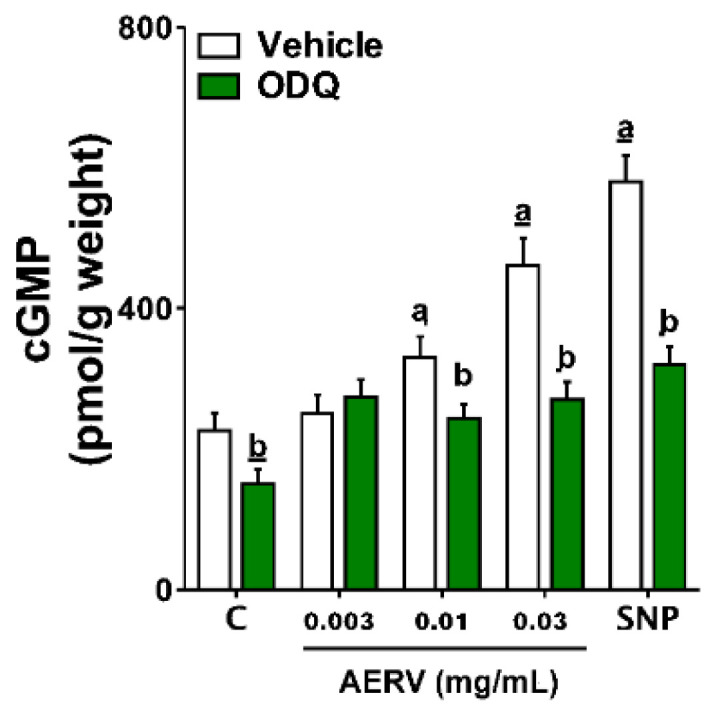
Vascular effects of AERV obtained from *Rudgea viburnoides* and the role of NO/cGMP pathway. In the absence and in presence of ODQ (100 μm), the intracellular cGMP levels from 2K1C-hypertensive rat aortic rings incubated with AERV (0.003, 0.01, and 0.03 mg/mL) or sodium nitroprusside (SNP) are expressed. The values are expressed as mean ± S.E.M. applying 8 to 10 preparations per group. (*p* < 0.05 vs. ^a^ control (C) or after incubation with ^b^ ODQ).

After the incubation of AERV (0.01 and 0.03 mg/mL) with the aortic rings of 2K1C-hypertensive rats, it was observed an increased the cGMP levels of approximately 39% and 81%, respectively, compared with basal levels. Although its co-incubation with ODQ (100 μm) abolished this effect. The cGMP levels were increased at circa 106% by NO-donor SNP, but the co-incubation with ODQ blocked the SNP-mediated increases in cGMP (Figure 6).

## 4. Discussion

A renovascular hypertension (RH) model was applied in our study, which was developed by Goldblatt [36]—i.e., 2K1C-model. This pathology is commonly produced by stenosis of the renal artery, which leads to the hypoperfusion of juxtaglomerular cells with sustained activation of the renin-angiotensin system (RAS). This problem accounts for only 1–2% of cases of hypertension in the adult population, but it is important in the secondary causes of hypertension in young people. Furthermore, this disease is common in patient with atherosclerosis and appears to be frequent in certain high-risk populations. The most indicated therapeutic procedures for HR treatment are to control of blood pressure levels, preventing ischemic nephropathy progression and biochemical and structural (vascular and cardiac) changes that lead to high morbidity and mortality rates [37].

Firstly, a progressive reduction of renal function in RH was observed. Progressive renal failure provoked a rise to the cardiorenal syndrome, which leads to heart and renal failures [38]. The volume and urinary electrolyte concentration are significantly reduced, and it is associated with increased serum creatinine levels, indicating a negative prognosis on renal function [39]. Here, 2K1C-hypertensive animals showed a significant time-dependent decline in renal function. At the end of 28 days, the renal function was markedly reduced, and the serum creatinine and urea levels were increased. Prolonged AERV treatment, especially at its highest doses, blocked these changes and produced similar responses to normotensive animals. Despite of the recognized hypotensive effects of MPL, a classic antihypertensive drug, it did not reverse the alterations presented by RH. Thus, MPL showed an inferior effectiveness compared to results of the AERV treatment.

Another important consequence of sustained activation of RAS during RH is vascular functional changes and cardiac remodeling induced by hypertension. Pressure overload induces cardiac remodeling, consisting of diastolic dysfunction and concentric left ventricular hypertrophy. These changes are the most common cardiac complications of hypertension, and a dilated cardiomyopathy is established with diastolic dysfunction and reduced ejection fraction [39]. These complications can be determined and diagnosed by electrocardiographic evaluation, measurement of the ventricular wall, or by atrial and ventricular hemodynamic parameters [40]. We observed electrocardiographic changes in the 2K1C-hypertensive rats, which are compatible to classic cardiac dysfunctions induced by hypertension. All the no-treated hypertensive rats revealed a significant P wave flattening, and QT prolongation. The changes in P-wave amplitude typically reflect atrial overload, hypertrophy or dilation, that is a frequent alteration in hypertension and stroke [41]. However, the QT interval is inversely proportional to the heart rate, this parameter is measured from the beginning of the QRS complex at the end of the T wave. In addition, the QT interval measured should be divided by the square root of the preceding RR interval for the correction of heart rate, and so this corrected measure is referred to the QTc interval. Although the QTc interval was normal, the animals were tachycardic, indicating they are more leaning to arrhythmias as a result from the prolonged QT interval [42]. These alterations are generally observed in hypertensive patients, especially if they show considerable morphological changes. The QT interval can be increased in severe concentric, and eccentric left ventricular hypertrophy [43]. If we look at our results, we see that animals with electrocardiographic abnormalities also present a significant thickening of the posterior ventricular wall, a fact that indicates an important correlation between hypertension, ventricular remodeling, and cardiac electrical changes. In the meantime, the efficacy of AERV was surprising, since in addition to reducing blood pressure levels and heart rate, it also prevented cardiac morphological and electrocardiographic changes 2K1C-hypertensive animals.

The relationship between hypertension and endothelial function is now well established [44]. Thus, the severity of hypertension can be positively associated with the degree of impairment of endothelial function [45]. Besides, it has been correlated to a complex and potentially bidirectional relationship between hypertension and endothelial dysfunction [46]. In fact, oxidative stress and vascular inflammation are central features of endothelial dysfunction. The changes in reactive oxygen species (ROS) production from mitochondrial NADPH oxidase seem to be one of the main issues involved in hypertensive patients with high levels of angiotensin II [47]. Hypertensive stimuli, such as high salt and angiotensin II, promote the ROS-production in the heart, kidney, and vasculature, and they contribute to endothelial dysfunction or others cardiovascular changes [48]. The RH model used in this study induced important changes in vascular redox status, leading to profound alterations in vascular reactivity of 2K1C-hypertensive rats.

The reduction of oxidative stress and vascular inflammation have been related to reverse hypertension-induced endothelial dysfunction [49]. An emerging question that arose after the analysis of the abovementioned data was how much the cardiorenal protection induced by AERV is a consequence of a direct ROS-reduction or in an indirect way via RAS inhibition. The indirect effects were evident after concluding that AERV has a significant inhibitory effect on plasma ACE. Classic ACE inhibitors or drugs that indirectly inhibit RAS—such as MPL, a beta-blocker—may have hypotensive and antioxidant effects [50,51]. Actually, we found an important reduction of lipoperoxides (LPO) in cardiac and vascular tissues of 2K1C-hypertensive rats. AERV was also able to effectively modulate the activity of SOD and CAT, two important enzymes that constitute the first line of antioxidant tissue defense.

We decided to investigate the direct activation of the NO/cGMP pathway by AERV, and thus clarifying if there was any antioxidant and cardioprotective effects induced by the extract independently of RAS inhibition. Interestingly, a notable increase was observed after exposure to AERV in intracellular levels of cGMP from the aortic smooth muscle cells of 2K1C-hypertensive rats that suggested an independent activation of the NO/cGMP pathway. The antioxidant and cardioprotective effects of NO are already well known and reported. Several drugs and natural products with antioxidant effects can attenuate NO inactivation by superoxide and other reactive oxygen species [52,53]. In these cases, the production of NO can be induced independently of eNOS-activity and this production may also be formed from nitrite, as reported for the administration of some natural products [53]. Nitrite has shown antihypertensive effects in 2K1C-hypertensive rats by inhibition of vascular NADPH oxidase with consequent reduction of ROS production [54].

A limitation of our study was not to show if there is any interrelation between ACE inhibitions by AERV with NO/cGMP pathway activation, but the modulation of the tissue redox state appears to be an important point of convergence. Furthermore, it has not been clear to us if there is any hierarchy of response between the different secondary metabolites found in the AERV. Our chemical study showed that AERV presents in its composition polyphenolic compounds (glycosylated flavonols and chlorogenic acids), as well as triterpenoid saponins, glycosylated triterpenes and glycosylated iridoids.

The polyphenol-rich plants have been reported as antihypertensive effects and these properties can occur via the NO-cGMP pathway and ACE inhibition [55]. Flavonoids, polyphenols widely found in plants, are recognized by their antioxidant, vasorelaxant, and antihypertensive properties, as well as benefits in cardiovascular diseases with cardio and nephroprotective effects [56,57,58,59]. Thus, these polyphenols have been commonly related to antihypertensive effects from vegetal extracts, as other polyphenols, such as chlorogenic acids [60,61]. Additionally, saponins have been reported as bioactive compounds from extracts, which have shown diuretic properties and action to reduce the arterial blood pressure [62,63].

## 5. Conclusions

Our study showed that prolonged treatment with AERV presents a significant cardiorenal protective effect in rats with renovascular hypertension, preserving the urine excretion and electrolyte levels and reducing the blood pressure and heart rates. The data obtained suggest the role of the NO/cGMP pathway activation associated with the inhibition of the angiotensin-converting enzyme. Thus, these results support the traditional uses of *R. viburnoides*.

## Figures and Tables

**Table 1:** Compounds annotated from aqueous extract of *Rudgea viburnoides* (AERV) by LC-DAD-MS/MS.

Peak	RT (min)	UV(nm)	MF	Positive Ion Mode (*m/z*)	Negative Ion Mode (*m/z*)		Compound	Metabolite Class	%
				MS [M+H]^+^	MS [M-H]^−^	MS/MS			
1	1.1	-	C_7_H_12_O_6_	193.0696	191.0534	191 (bp), 173	Quinic acid	Carboxylic acid	18.34
2	1.1	-	C_12_H_22_O_11_	-	341.1095	191(bp)	di-*O*-hexoside	Primary metabolite	4.36
3	1.5	-	C_6_H_8_O_7_	193.0367	191.0209	-	Citric acid	Carboxylic acid	1.31
4	2.7	-	C_16_H_20_O_10_	373.1113	371.0991	191, 173 (bp)	Glycosylated iridoid	Iridoid	0.31
5	5.9	295 ^sh^, 325	C_16_H_18_O_9_	355.1006	353.0873	191 (bp), 179	3-*O*-*E*-caffeoylquinic acid ^st^	Chlorogenic acid	3.66
6	9.9	-	C_18_H_24_O_12_	-	431.1195	251, 165 (bp)	Asperulosidic acid	Iridoid	4.67
7	10.5	295 ^sh^, 325	C_16_H_18_O_9_	355.1021	353.0873	191 (bp), 179	5-*O*-*E*-caffeoylquinic acid ^st^	Chlorogenic acid	2.48
8	11.3	-	C_18_H_22_O_11_	415.1223	413.1084	191, 147 (bp)	Asperuloside	Iridoid	11.98
9	18.7	265, 355	C_27_H_30_O_16_	611.1595	609.1472	300 (bp), 301, 271, 255, 243	*O*-hexosyl-deoxyhexosyl quercetin	Flavonol	3.87
10	20.0	-	C_24_H_42_O_11_	507.2822	505.2655	191 (bp)	Unknown	-	1.81
11	20.8	265, 346	C_27_H_30_O_15_	595.1644	593.1521	327, 285 (bp), 284, 255, 277, 162	*O*-hexosyl-deoxyhexosyl kaempferol	Flavonol	9.26
12	25.0	-	C_36_H_58_O_12_	683.3967	681.3823	519 (bp), 501, 407, 207	Trachelosperoside B-1 or E-1	Glycosylated triterpene	14.40
13	25.1	-	C_36_H_58_O_12_	683.3981	681.3832	519 (bp), 501, 489, 457, 407, 207	Trachelosperoside B-1 or E-1	Glycosylated triterpene	12.84
14	26.6	-	C_36_H_58_O_12_	667.4027	665.3757	503 (bp), 441, 409	Glycosylated triterpene (Arjunoglucoside I)	Glycosylated triterpene	2.25
15	32.1	-	C_53_H_84_O_23_	1089.5298	1087.5303	925 (bp), 793, 631, 613, 569, 469, 455, 353, 161	*O*-glucoronyl-hexosyl-pentosyl *O*-hexosyl triterpene	Triperpenoid saponin	2.09
16	32.2	-	C_48_H_76_O_19_	957.5026	955.4914	793 (bp), 749, 731, 631, 613, 569, 551, 455, 337, 179	*O*-glucoronyl-hexosyl *O*-hexosyl triterpene	Triperpenoid saponin	6.37

MF: molecular formula; RT: retention time; ^sh^: shoulder; bp: base peak; ^st^: confirmed by injection of authentic standard; %: relative percentage of area obtained in negative ion mode.

**Table 2:** Effects on urinary volume and electrolyte excretion, pH, and density of AERV obtained from *Rudgea viburnoides* on first day of treatment.

Group	Urinary Volume (mL/100 g/8 h)	El_Na+_ (µEq/min/100 g)	El_k+_ (µEq/min/100 g)	El_Ca2+_ (µEq/min/100 g)	El_Cl−_ (µEq/min/100 g)	pH	Density
Sham	3.771 ± 0.188	1.694 ± 0.108	0.571 ± 0.029	0.021 ± 0.001	1.902 ± 0.113	8.5 ± 0.04	1015 ± 0.84
C-	4.517 ± 0.219	0.826 ± 0.006 ^b^	0.546 ± 0.010	0.012 ± 0.002 ^b^	0.981± 0.016 ^b^	8.3 ± 0.02	1010 ± 0.28 ^b^
AERV 30 (mg/kg)	3.665 ± 0.021	0.887 ± 0.087 ^b^	0.615 ± 0.032	0.007 ± 0.001 ^b^	1.021 ± 0.029 ^b^	8.7 ± 0.13	1015 ± 0.42
AERV (100 mg/kg)	3.831 ± 0.445	0.878 ± 0.057 ^b^	0.614 ± 0.045	0.012 ± 0.001 ^b^	1.026 ± 0.074 ^b^	8.7 ± 0.15	1015 ± 0.42
AERV (300 mg/kg)	3.279 ± 0.129	1.473 ± 0.054 ^a^	0.554 ±0.018	0.029 ± 0.001 ^a^	1.653 ± 0.025 ^a^	8.6 ± 0.05	1016 ± 1.52
MPL (25 mg/kg)	4.378 ± 0.223	1.651 ± 0.082 ^a^	0.520 ± 0.069	0.022 ± 0.003 ^a^	1.791 ± 0.038 ^a^	8.1 ± 0.17	1014 ± 1.15

The values are expressed as mean ± S.E.M. In each group, 8 to 10 animals were used, and the comparisons were performed with the negative control (C-; ^a^ *p* < 0.05) or Sham-operated group (^b^ *p* < 0.05) applying one-way ANOVA followed by Bonferroni’s test. El: excreted load; MPL: metoprolol.

**Table 3:** Effects on urinary volume and electrolyte excretion, pH and density of AERV obtained from *Rudgea viburnoides* on seventh day of treatment.

Group	Urinary Volume (mL/100 g/8 h)	El_Na+_ (µEq/min/100 g)	El_k+_ (µEq/min/100 g)	El_Ca2+_ (µEq/min/100 g)	El_Cl−_ (µEq/min/100 g)	pH	Density
Sham	5.272 ± 0.224	1.207 ± 0.087	0.591 ± 0.031	0.011 ± 0.001	1.348 ± 0.194	7.8 ± 0.06	1007 ± 0.42
C-	3.862 ± 0.613 ^b^	1.261 ± 0.124	0.597 ± 0.034	0.013 ± 0.003	1.344 ± 0.127	7.7 ± 0.05	1013 ± 0.57 ^b^
AERV 30 (mg/kg)	3.661 ± 0.208 ^b^	1.178 ± 0.026	0.570 ± 0.011	0.009 ± 0.001	1.319 ± 0.122	7.8 ± 0.07	1017 ± 1.11 ^b^
AERV (100 mg/kg)	2.939 ± 0.396 ^b^	0.906 ± 0.096	0.438 ± 0.022	0.009 ± 0.001	0.971 ± 0.106	7.8 ± 0.11	1017 ± 1.83 ^b^
AERV (300 mg/kg)	3.513 ± 0.334 ^b^	0.702 ± 0.268	0.396 ± 0.099	0.008 ± 0.004	0.803 ± 0.314	7.6 ± 0.14	1006 ± 2.30 ^a^
MPL (25 mg/kg)	4.043 ± 0.333	1.181 ± 0.140	0.540 ± 0.078	0.009 ± 0.006	1.295 ± 0.182	7.7 ± 0.02	1008 ± 0.57 ^a^

The values are expressed as mean ± S.E.M. In each group, 8 to 10 animals were used, and the comparisons were performed with the negative control (C-; ^a^ *p* < 0.05) or Sham-operated group (^b^ *p* < 0.05) applying one-way ANOVA followed by Bonferroni’s test. El: excreted load; MPL: metoprolol.

**Table 4:** Effects on urinary volume and electrolyte excretion, pH, and density of AERV obtained from *Rudgea viburnoides* on fourteenth day of treatment.

Group	Urinary Volume (mL/100 g/8 h)	El_Na+_ (µEq/min/100 g)	El_k+_ (µEq/min/100 g)	El_Ca2+_ (µEq/min/100 g)	El_Cl−_ (µEq/min/100 g)	pH	Density
Sham	4.319 ± 0.399	1.363 ± 0.101	0.635 ± 0.057	0.034 ± 0.002	1.853 ± 0.124	7.8 ± 0.02	1016 ± 0.96
C-	3.389 ± 0.032 ^b^	0.846 ± 0.049 ^b^	0.809 ± 0.101	0.017 ± 0.003 ^b^	1.376 ± 0.096 ^b^	7.8 ± 0.14	1018 ± 0.80
AERV 30 (mg/kg)	3.139 ± 0.186 ^b^	0.893 ± 0.028 ^b^	0.641 ± 0.049	0.018 ± 0.002 ^b^	1.340 ± 0.016 ^b^	7.7 ± 0.20	1018 ± 1.23
AERV (100 mg/kg)	2.794 ± 0.407 ^b^	1.193 ± 0.141	0.658 ± 0.076	0.012 ± 0.002 ^b^	1.003 ± 0.153 ^b^	7.6 ± 0.16	1015 ± 0.73
AERV (300 mg/kg)	4.350 ± 0.164 ^a^	1.319 ± 0.023 ^a^	0.796 ± 0.129	0.040 ± 0.009 ^a^	1.807 ± 0.082 ^a^	7.7 ± 0.02	1016 ± 0.28
MPL (25 mg/kg)	3.975 ± 0.050 ^a^	1.169 ± 0.118	0.652 ± 0.009	0.009 ± 0.002 ^b^	1.190 ± 0.134 ^b^	7.6 ± 0.08	1017 ± 1.15

The values are expressed as mean ± S.E.M. In each group, 8 to 10 animals were used, and the comparisons were performed with the negative control (C-; ^a^ *p* < 0.05) or Sham-operated group (^b^ *p* < 0.05) applying one-way ANOVA followed by Bonferroni’s test. El: excreted load; MPL: metoprolol.

**Table 5:** Effects on urinary volume and electrolyte excretion, pH, and density of AERV obtained from *Rudgea viburnoides* on twenty-first day of treatment.

Group	Urinary Volume (mL/100 g/8 h)	El_Na+_ (µEq/min/100 g)	El_k+_ (µEq/min/100 g)	El_Ca2+_ (µEq/min/100g)	El_Cl−_ (µEq/min/100 g)	pH	Density
Sham	5.111 ± 0.117	1.319 ± 0.144	0.667 ± 0.030	0.020 ± 0.001	1.486 ± 0.041	7.2 ± 0.12	1012 ± 0.55
C-	3.209 ± 0.422 ^b^	1.157 ± 0.182	0.419 ± 0.069 ^b^	0.007 ± 0.004 ^b^	1.279 ± 0.196	7.3 ± 0.12	1018 ± 1.15 ^b^
AERV 30 (mg/kg)	3.143 ± 0.040 ^b^	1.192 ± 0.011	0.523 ± 0.015	0.027 ± 0.001 ^a^	1.422 ± 0.015	7.6 ± 0.17	1022 ± 0.73 ^b^
AERV (100 mg/kg)	3.712 ± 0.099 ^b^	1.295 ± 0.028	0.565 ± 0.013	0.018 ± 0.002 ^a^	1.417 ± 0.052	7.6 ± 0.19	1017 ± 1.11 ^b^
AERV (300 mg/kg)	4.387 ± 0.264 ^a^	0.899 ± 0.169	0.781 ± 0.013 ^a^	0.041 ± 0.002 ^a^	1.344 ± 0.060	7.3 ± 0.11	1012 ± 1.73 ^a^
MPL (25 mg/kg)	3.062 ± 0.022 ^b^	0.880 ± 0.182	0.495 ± 0.099	0.009 ± 0.003 ^b^	0.892 ± 0.213 ^b^	7.3 ± 0.34	1019 ± 0.28 ^b^

The values are expressed as mean ± S.E.M. In each group, 8 to 10 animals were used, and the comparisons were performed with the negative control (C-; ^a^ *p* < 0.05) or Sham-operated group (^b^ *p* < 0.05) applying one-way ANOVA followed by Bonferroni’s test. El: Excreted load; MPL: Metoprolol.

**Table 6:** Effects on urinary volume and electrolyte excretion, pH, and density of AERV obtained from *Rudgea viburnoides* on twenty-eighth day of treatment.

Group	Urinary Volume (mL/100 g/8 h)	El_Na+_ (µEq/min/100 g)	El_k+_ (µEq/min/100 g)	El_Ca2+_ (µEq/min/100 g)	El_Cl−_ (µEq/min/100 g)	pH	Density
Sham	4.196 ± 0.098	1.348 ± 0.086	0.639 ± 0.026	0.063 ± 0.005	1.532 ± 0.081	7.4 ± 0.17	1015 ± 0.91
C-	3.350 ± 0.064 ^b^	0.860 ± 0.022 ^b^	0.680 ± 0.010	0.020 ± 0.001 ^b^	1.039 ± 0.027 ^b^	7.6 ± 0.18	1019 ± 0.57 ^b^
AERV 30 (mg/kg)	3.504 ± 0.142 ^b^	1.311 ± 0.102	0.681 ± 0.045	0.022 ± 0.003 ^b^	1.527 ± 0.085 ^a^	7.7 ± 0.17	1018 ± 0.42 ^b^
AERV (100 mg/kg)	3.449 ± 0.125 ^b^	1.296 ± 0.071	0.608 ± 0.001	0.015 ± 0.001 ^b^	1.413 ± 0.077 ^a^	7.6 ± 0.14	1014 ± 0.55 ^a^
AERV (300 mg/kg)	3.927 ± 0.192 ^a^	1.289 ± 0.079	0.667 ± 0.026	0.037 ± 0.016	1.465 ± 0.098 ^a^	7.5 ± 0.12 ^a^	1015 ± 0.57 ^a^
MPL (25 mg/kg)	3.433 ± 0.215 ^b^	1.274 ± 0.159	0.678 ± 0.106	0.016 ± 0.003 ^b^	1.165 ± 0.171	7.3 ± 0.11	1017 ± 2.30

The values are expressed as mean ± S.E.M. In each group, 8 to 10 animals were used, and the comparisons were performed with the negative control (C-; ^a^ *p* < 0.05) or Sham-operated group (^b^ *p* < 0.05) applying one-way ANOVA followed by Bonferroni’s test. El: excreted load; MPL: metoprolol.

**Table 7:** Effects on serum Na^+^, K^+^, urea, creatinine, and plasmatic ACE activity of AERV obtained from *Rudgea viburnoides* on twenty-eighth day of treatment.

Group	Na^+^ (mmol/L)	K^+^ (mmol/L)	Urea (mg/dL)	Creatinine (mg/dL)	ACE Activity (mmol/min/mL)
Sham	126.5 ± 1.21	5.81 ± 0.22	57.32 ± 0.93	0.32 ± 0.02	85 ± 10.10
C-	130.2 ± 1.86	6.15 ± 0.35	85.80 ± 2.75 ^b^	0.67 ± 0.03 ^b^	155 ± 13.22 ^b^
AERV (30 mg/kg)	131.7 ± 1.99	6.22 ± 0.12	77.08 ± 1.56 ^b^	0.59 ± 0.02 ^b^	121 ± 9.11 ^b^
AERV (100 mg/kg)	129.9 ± 1.23	6.13 ± 0.51	56.63 ± 1.17	0.33 ± 0.01	98 ± 10.24 ^a^
AERV (300 mg/kg)	126.0 ± 2.03	5.77 ± 0.22	57.36 ± 1.23	0.35 ± 0.03	90 ± 12.11 ^a^
MPL (25 mg/kg)	129.1 ± 1.92	5.41 ± 0.21	58.22 ± 3.21	0.37 ± 0.05	100 ± 11.08 ^a^

The values are expressed as mean ± S.E.M. In each group, 8 to 10 animals were used, and the comparisons were performed with the negative control (C-; ^a^ *p* < 0.05) or Sham-operated group (^b^ *p* < 0.05) applying one-way ANOVA followed by Bonferroni’s test. El: excreted load; MPL: metoprolol.

**Table 8:** Effects on tissue redox status of AERV obtained from *Rudgea viburnoides*.

Parameter	Sham	C-	AERV (30 mg/kg)	AERV (100 mg/kg)	AERV (300 mg/kg)	MPL (25 mg/kg)
Heart						
SOD	40.57 ± 1.61	30.54 ± 0.61 ^b^	34.89 ± 1.49 ^b^	38.03 ± 1.23 ^a^	40.46 ± 1.40 ^a^	34.84 ± 1.84 ^b^
Cat	45.10 ± 4.33	25 ± 5.15 ^b^	52 ± 6.68 ^a^	45 ± 3.61 ^a^	55 ± 9.21 ^a^	28 ± 75.35 ^b^
LPO	16.48 ± 0.97	25.17 ± 0.35 ^b^	18.10 ± 0.65	17.99 ± 0.73	17.42 ± 0.59 ^a^	17.93 ± 0.37
Aorta						
SOD	71.73 ± 5.00	62.37 ± 2.55	67.48 ± 1.36	68.61 ± 1.37	69.64 ± 1.73	62.83 ± 1.53
Cat	61 ± 3.79	41 ± 4.58 ^b^	52 ± 5.79	58 ± 4.38 ^a^	77 ± 4.25 ^a^	49 ± 4.31 ^b^
LPO	6.90 ± 0.53	8.54 ± 0.38 ^b^	7.61 ± 0.41	7.29 ± 0.41	6.91 ± 0.47 ^a^	7.22 ± 0.33

The values are expressed as mean ± S.E.M. In each group, 8 to 10 animals were used, and the comparisons were performed with the negative control (C-; ^a^ *p* < 0.05) or Sham-operated group (^b^ *p* < 0.05) applying one-way ANOVA followed by Bonferroni’s test. MPL: metoprolol. SOD: superoxide dismutase (Unit of SOD/mg of protein); Cat: catalase (unit of SOD/mg of protein); LPO: lipoperoxides (nmol hydroperoxides/mg of protein).

## Data Availability

Data presented in this study are included within the article.

## Supplementary Materials

The following are available online at https://www.mdpi.com/article/10.3390/pharmaceutics13101579/s1, Figure S1: Representative cross-sections of the heart, liver, and kidney in the control and AERV-treated rats. H&E stain. 40 X, Table S1: Effects of acute administration of the AERV (30, 300, and 2000 mg/kg) on body weight and food and water consumption at 14th day.
