# Supplementary material for: Prolonged Administration of Rudgea viburnoides (Cham.) Benth. Prevents Impairment of Redox Status, Renal Dysfunction, and Cardiovascular Damage in 2K1C-Hypertensive Rats by Inhibiting ACE Activity and NO-GMPC Pathway Activation
Source: Pharmaceutics. 2021 Sep 28;13(10):1579. doi: 10.3390/pharmaceutics13101579 (PMC8537958; doi:10.3390/pharmaceutics13101579)
Supplement: Supplementary file 1 [file pharmaceutics-13-01579-s001.zip › pharmaceutics-1384341-supplementary.pdf]

pharmaceutics

MDPI

Supplementary Material

Prolonged Administration of *Rudgea viburnoides* (Cham.) Benth. Prevents Impairment of Redox Status, Renal Dysfunction, and Cardiovascular Damage in 2K1C-Hypertensive Rats by Inhibiting ACE Activity and NO-GMPC Pathway Activation

Fernanda Viana Paulin [1], Rhanany Alan Calloi Palozi [2], Bethânia Rosa Lorençone [2], Arthur Ladeira Macedo [1], Lucas Pires Guarnier [2], Cleide Adriane Signor Tirloni [2], Paulo Vitor Moreira Romão [2], Arquimedes Gasparotto Junior [2] and Denise Brentan Silva [1,*]

[1] Laboratório de Produtos Naturais e Espectrometria de Massas (LaPNEM), Faculdade de Ciências Farmacêuticas, Alimentos e Nutrição (FACFAN), Universidade Federal do Mato Grosso do Sul, Campo Grande, 79070-900, Brazil; fernanda@movecor.com.br (F.V.P.); arthur.ladeira@ufms.br (A.L.M.)
[2] Laboratório de Farmacologia Cardiovascular (LaFaC), Faculdade de Ciências da Saúde, Universidade Federal da Grande Dourados, Dourados, 79825-070, Brazil; palozirhanany@gmail.com (R.A.C.P.); bethaniarosalorencone@hotmail.com (B.R.L.); rannier.andrade@outlook.com (L.P.G.); cleide.4132@gmail.com (C.A.S.T.); paulovitor_moreiraromao@hotmail.com (P.V.M.R.); arquimedesjunior@ufgd.edu.br (A.G.J.)
* Correspondence: denise.brentan@ufms.br

Citation: Paulin, F.V.; Palozi, R.A.C.; Lorençone, B.R.; Macedo, A.L.; Guarnier, L.P.; Tirloni, C.A.S.; Romão, P.V.M.; Gasparotto Junior, A.; Silva, D.B. Prolonged Administration of *Rudgea viburnoides* (Cham.) Benth. Prevents Impairment of Redox Status, Renal Dysfunction and Cardiovascular Damage in 2K1C-Hypertensive Rats by Inhibiting ACE Activity and NO-GMPC Pathway Activation. *Pharmaceutics* **2021**, *13*, 1579. https://doi.org/10.3390/pharmaceutics13101579

Academic Editor: Yasumasa Ikeda

Received: 1 September 2021
Accepted: 26 September 2021
Published: 28 September 2021

Publisher's Note: MDPI stays neutral with regard to jurisdictional claims in published maps and institutional affiliations.

Copyright: © 2021 by the authors. Licensee MDPI, Basel, Switzerland. This article is an open access article distributed under the terms and conditions of the Creative Commons Attribution (CC BY) license (http://creativecommons.org/licenses/by/4.0/).

Table S1. Effects of acute administration of the AERV (30, 300, and 2000 mg/kg) on body weight and food and water consumption at 14th day.

Parameter	Naïve	AEBP (30 mg/kg)	AEBP (300 mg/kg)	AEBP (2000 mg/kg)
Food consumption (mL)	18.27 ± 1.20	17.11 ± 1.17	18.01 ± 1.12	17.86 ± 1.29
Water consumption (g)	9.17 ± 1.21	9.02 ± 1.11	8.97 ± 0.99	9.04 ± 0.89
Body weight (g)	226.33 ± 18.12	224.15 ± 15.26	231.12 ± 19.11	221.19 ± 17.33

Statistical analyses were performed by Student's t-test. Values are expressed as mean ± SEM (standard error of the mean) of 8 animals per group

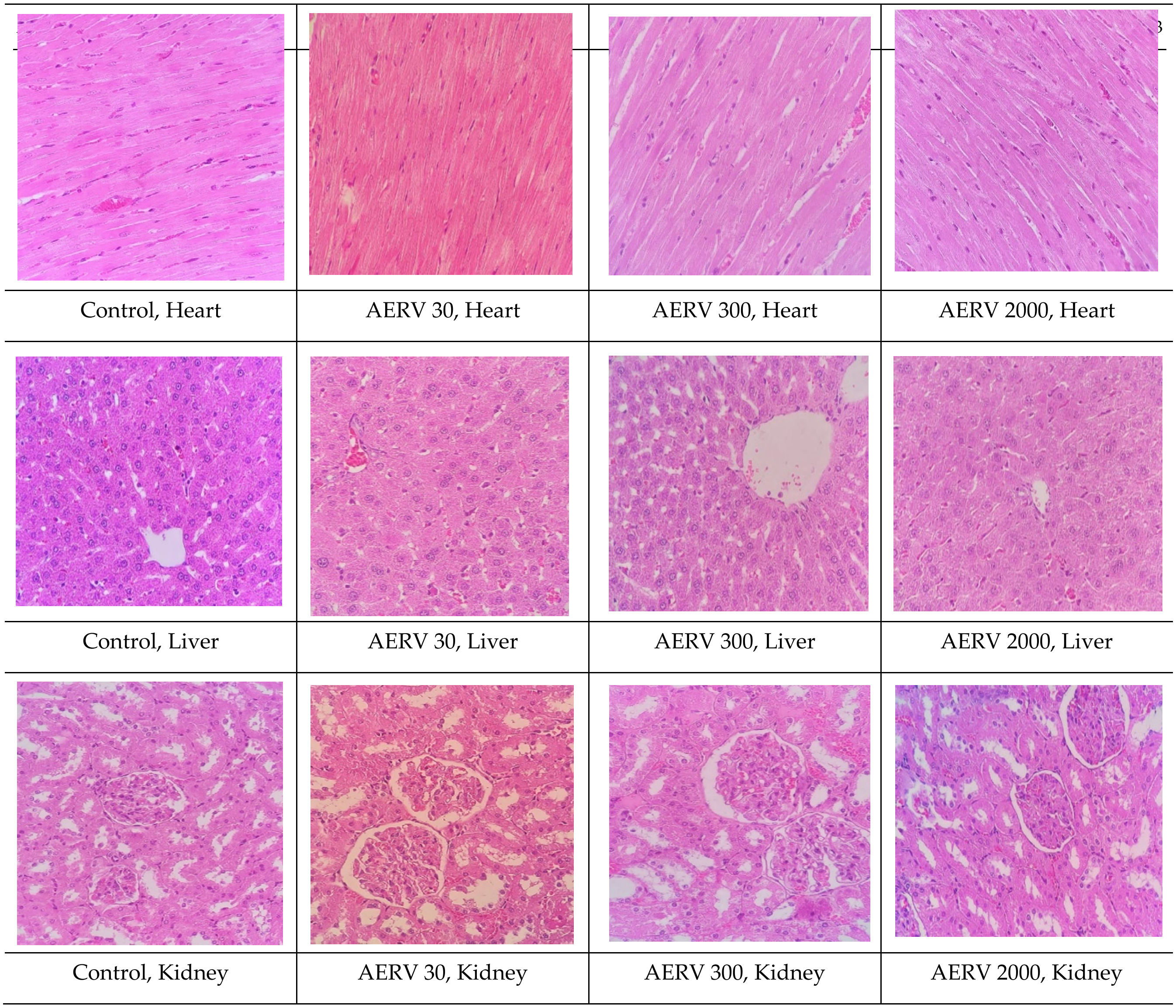

Figure S1. Representative cross-sections of the heart, liver, and kidney in the control and AERV-treated rats. H&E stain. 40 X.
